# Deaths in Philadelphia from July 20th to August, 24th, 1850

**Published:** 1850-09

**Authors:** James Aitken Meigs

**Affiliations:** Student of Medicine


*Deaths in Philadelphia from July 20th to Aug. 24th*, 1850. Reported by Mr. James Aitken Meigs, Student of Medicine.

| Diseases. | Ad'ts | Chil. | Diseases. | Ad'ts | Chil. |
| --- | --- | --- | --- | --- | --- |
| Abscess of knee, . . | 1 | 0 | Enlargement of heart, . | 2 | 3 |
| " liver, . . | 1 | 0 | Epilepsy, . . . . | 1 | 1 |
| " lumbar vertebræ . | 1 | 0 | Erysipelas, . . . | 0 | 2 |
| Adynamia, . . . | 1 | 1 | Exhaustion, . . . | 3 | 0 |
| Anæmia, . . . | 2 | 2 | Fever, . . . . . | 0 | 2 |
| Apoplexy, . . . | 12 | 0 | " bilious, . . | 1 | 1 |
| Asthma, . . . . | 4 | 1 | " congestive, . . | 2 | 1 |
| Asphyxia, . . . | 0 | 1 | " hectic, . . | 0 | 1 |
| Anuria, . . . . | 0 | 2 | " intermittent, . | 0 | 1 |
| Burns, . . . | 1 | 0 | " puerperal, . . | 4 | 0 |
| Cancer, . . . . | 1 | 0 | " scarlet, . . | 0 | 13 |
| " breast, . . | 1 | 0 | " remittent, . . | 0 | 3 |
| " mouth, . . | 0 | 2 | " typhoid, . . | 4 | 5 |
| " stomach, . | 4 | 0 | " typhus, . . | 3 | 2 |
| " uterus, . . | 3 | 0 | Fracture, . . . | 1 | 0 |
| Cancrum oris, . . | 0 | 1 | " of pelvis, . . | 1 | 0 |
| Casualties, . . . | 4 | 3 | Fistula, . . . . | 1 | 0 |
| Cholera, . . . . | 2 | 0 | Gangrene, . . . | 1 | 0 |
| Cholera infantum, . . | 0 | 198 | Gout, . . . . | 1 | 0 |
| " morbus, . . | 5 | 0 | Hæmoptysis, . . . | 1 | 0 |
| Compression of brain, . | 1 | 0 | Hemorrhage from brain, . | 1 | 3 |
| Congestion of lungs, . | 2 | 3 | " uterus, . | 1 | 0 |
| " brain, . | 8 | 11 | Inflammation of brain, . | 2 | 29 |
| Convulsions, . . . | 0 | 68 | " bronchi, . . | 1 | 10 |
| " puerperal, . | 1 | 0 | " breast, . . | 1 | 0 |
| Cramp, . . . . | 1 | 0 | " cerebrum, . | 0 | 2 |
| Croup, . . . . | 0 | 8 | " kidneys, . . | 1 | 0 |
| Coup de soleil, . . | 3 | 0 | " liver, . . | 4 | 2 |
| Cyanosis, . . . | 0 | 4 | " lungs, . . | 10 | 10 |
| Debility, general, . . | 10 | 26 | " peritoneum, . | 2 | 2 |
| Diarrhœa, . . . | 16 | 48 | " pleura, . . | 1 | 0 |
| Disease of brain, . . | 1 | 8 | " larynx, . . | 1 | 1 |
| " elbow joint, . | 1 | 0 | " stom. & bowels, | 12 | 18 |
| " heart, . . | 6 | 1 | " throat, . . | 0 | 1 |
| " hip, . . | 0 | 1 | Inanition, . . . | 2 | 6 |
| " kidneys, . | 1 | 0 | Intemperance, . . | 5 | 0 |
| " liver, . . | 1 | 0 | Jaundice, . . . | 2 | 2 |
| " lungs, . . | 1 | 0 | Malformation, . . | 0 | 5 |
| " spine, . . | 0 | 3 | " of heart, . | 0 | 1 |
| " spleen, . . | 1 | 0 | Mania-a-potu, . . | 4 | 0 |
| " scrotum, . | 1 | 0 | Marasmus, . . . | 3 | 47 |
| " stomach and bowels, | 4 | 2 | Measles, . . . | 0 | 6 |
| Dropsy, . . . | 14 | 0 | Mortification of bowels, . | 3 | 0 |
| " of breast, . . | 2 | 3 | Old age, . . . . | 25 | 0 |
| " head, . . | 0 | 50 | Palsy, . . . . | 7 | 0 |
| Drowned, . . . | 13 | 9 | Pertussis, . . . | 0 | 23 |
| Dysentery, . . . | 54 | 66 | Phthisis pulmonalis, . | 72 | 12 |
| Effusion on brain, . . | 2 | 11 | Poisoning, . . . | 1 | 0 |
| " lungs, . . | 0 | 1 | Purpura hemorrhagica, . | 0 | 3 |

| Diseases. | Ad'ts | Chil. | Diseases. | Ad'ts | Chil. |
|---|---|---|---|---|---|
| Scirrhus of liver, . . | 2 | 0 | Teething, . . . | 0 | 2 |
| " uterus . . | 1 | 0 | Tetanus, . . . | 0 | 1 |
| Scrofula, . . . | 1 | 5 | Tuberculosis, . . . | 1 | 1 |
| Small pox, . . . | 1 | 3 | Ulceration of larynx, . | 0 | 1 |
| Softening of brain, . . | 1 | 0 | " throat, . | 0 | 1 |
| " stomach, . | 0 | 2 | Unknown, . . . | 8 | 16 |
| Still born, . . . | 0 | 38 | Varicella, . . . | 0 | 1 |
| Suicide, . . . . | 4 | 0 | Violence, . . . | 2 | 1 |
| Tabes mesenterica, . | 0 | 5 | | | |
| | | | | 395 | 828 |

Total, . . . . . . . . . . . 1223

Of the foregoing the ages were as follows:—

| | | | | |
|---|---|---|---|---|
| Under 1 year, | | - | - - | 427 |
| From 1 | to | - | 2, - - | 217 |
| 2 | - | - | 5, - - | 100 |
| 5 | - | - | 10, - - | 37 |
| 10 | - | - | 15, - - | 25 |
| 15 | - | - | 20, - - | 22 |
| 20 | - | - | 30, - - | 81 |
| 30 | - | - | 40, - - | 99 |
| 40 | - | - | 50, - - | 65 |
| 50 | - | - | 60, - - | 41 |
| 60 | - | - | 70, - - | 54 |
| 70 | - | - | 80, - - | 31 |
| 80 | - | - | 90, - - | 17 |
| 90 | - | - | 100, - - | 7 |
| | | | | 1223 |

Included in this number, are 84 from the Almshouse, 30 from the surrounding country, and 70 people of color.

